# Physiological and Biochemical Properties of Cotton Seedlings in Response to Cu^2+^ Stress

**DOI:** 10.3390/cimb45050258

**Published:** 2023-05-05

**Authors:** Hao Zhou, Ke-Hai Zhou, Gang Zhao, Pei-Pei Wang, Dai-Gang Yang, Xiong-Feng Ma, Jun-Shan Gao

**Affiliations:** 1 School of Life Sciences, Anhui Agricultural University, Hefei 230036, China; 2 Institute of Cotton Research, Chinese Academy of Agricultural Science, Anyang 455000, China

**Keywords:** copper, cotton, tolerance, antioxidants, reactive oxygen species

## Abstract

Copper(II) (Cu^2+^) is essential for plant growth and development. However, high concentrations are extremely toxic to plants. We investigated the tolerance mechanism of cotton under Cu^2+^ stress in a hybrid cotton variety (Zhongmian 63) and two parent lines with different Cu^2+^ concentrations (0, 0.2, 50, and 100 μM). The stem height, root length, and leaf area of cotton seedlings had decreased growth rates in response to increasing Cu^2+^ concentrations. Increasing Cu^2+^ concentration promoted Cu^2+^ accumulation in all three cotton genotypes’ roots, stems, and leaves. However, compared with the parent lines, the roots of Zhongmian 63 were richer in Cu^2+^ and had the least amount of Cu^2+^ transported to the shoots. Moreover, excess Cu^2+^ also induced changes in cellular redox homeostasis, causing accumulation of hydrogen peroxide (H_2_O_2_) and malondialdehyde (MDA). Conversely, antioxidant enzyme activity increased, while photosynthetic pigment content decreased. Our findings indicated that the hybrid cotton variety fared well under Cu^2+^ stress. This creates a theoretical foundation for the further analysis of the molecular mechanism of cotton resistance to copper and suggests the potential of the large-scale planting of Zhongmian 63 in copper-contaminated soils.

## 1. Introduction

Heavy metal contamination is a potential environmental issue worldwide due to increasing levels caused by both natural and manufactured activities [1]. Copper(II) (Cu^2+^) is an essential micronutrient for biological functions, as a constituent of the enzymes and proteins necessary for plant growth and development [2]. Research proved that 5–30 mg/kg Cu^2+^ is ideal for plant tissue growth [3,4]. When deficient, the visible symptoms include stunted growth, chlorosis of young leaves, losses in biomass and fruit yield, and ultimately the death of the plant [5,6]. However, Cu^2+^ in excess concentrations is also potentially harmful. Excess accumulation can destabilize membrane integrity, decrease photosynthesis, and alter enzyme activity, which result in growth inhibition and other detrimental effects on the primary production and survival of plants [7,8]. Moreover, Cu^2+^ disrupts key physiological processes and the metabolic functions of essential elements, thereby causing cellular redox imbalance and oxidative stress [9,10]. In addition, Cu^2+^ at higher concentrations causes the overproduction of reactive oxygen species (ROS) and reactive nitrogen species (RNS), which are cytotoxic and impair important cell compounds [6,11]. In response to Cu^2+^ toxicity, plants use homeostatic mechanisms to circumvent heavy metal toxicity through metal exclusion, immobilization in the cell wall, metal compartmentation, and binding of heavy metal by strong ligands [12,13]. In response to ROS production, both enzymatic and non-enzymatic antioxidant scavengers such as peroxidase (POD), superoxide dismutase (SOD), catalases (CAT), glutathione (GHS), ascorbate (ASA), and proline are highly activated to combat the oxidative injuries caused by heavy metal toxicity [14]. GSH, for example, is thought to play multiple anti-metal toxicity roles by reducing metal uptake and chelating metal ions [15,16]. Proline was found to reduce harmful heavy metal toxicity by acting as a hydroxyl radical scavenger in the cytoplasm [17]. Furthermore, POD, SOD, and CAT were found in high concentrations in response to heavy metal scavenging [17,18,19]. Similarly, in response to heavy metal toxicity, sugars can function as ROS eliminators or cell signals in response to metal toxicity in plants [19]. 

In China, a recent report showed that heavy metal contamination in cultivated farmlands exceeds 20 million hm^2^, and Cu^2+^ is a vital metal pollutant [20]. Cotton (*Gossypium hirsutum L*.) is a well-known economic crop that is grown all over the world, which is highly cultivated in farm areas in China [21]. Therefore, it faces a significant threat by Cu^2+^ toxicity [22]. Cu^2+^ toxicity and tolerance in plant species were extensively studied, including in Arabidopsis [23], fenugreek [24], and riparian plant species [25]. In cotton, foliar application of Cu^2+^ was found to significantly affect the lint percentage and fiber properties [26]. Although cotton is resistant to heavy metals and other abiotic stresses, the non-edible characteristics of cotton fiber give it the ability to restore soil quality when grown in heavily polluted areas [20]. However, no comprehensive study has been conducted on the specific toxic effects and responses to Cu^2+^ of cotton seedlings. As a result, the current study aimed to identify Cu^2+^ toxicity syndrome in two parental lines and their hybrid variety as well as further investigate the mechanisms of Cu^2+^ tolerance in cotton. Primarily, we observed the physiological and biochemical properties of cotton seedling responses to Cu^2+^ stress among two cotton parental lines (9053 and sGK9708) and their hybrid variety (Zhongmian 63). To achieve the purpose of this study, the cotton seedlings were exposed to CuSO_4_ at different concentrations to find the physio-molecular changes that occurred in the different genotypes. In addition, biomolecules, including POD and GHS, and ROS accumulation were assessed in the cotton seedlings in response to Cu^2+^ toxicity.

## 2. Materials and Methods

### 2.1. Plant Materials, Growth Condition, and Treatments

The glasshouse experiment was conducted using a conventional upland cotton line “9053” as the maternal parent, a new insect-resistant cotton variety improved line “sGK970” as the male parent, and their hybrid variety, Zhongmian 63, which is a bivalent transgenic insect-resistant cotton hybrid F1 generation and is planted in a large area of Yangtze River Basin, especially in Anhui Province and Hubei Province. Uniformly sized seeds were surface sterilized with a 1% (*v*/*v*) sodium hypochlorite solution for 20 min, washed with distilled water three times, and left for water imbibition for 24 h before growth in a sand culture medium. Seed germination occurred for 5 days at 28 °C in the dark. On the 6th day, uniform seedlings were transferred to hydroponic media and allowed to acclimatize for 10 days. The nutrient solution composition was 20 µM H_3_BO_3_, 1 µM ZnSO_4_•⸳7H_2_O, 0.2 µM CuSO_4_⸳5H_2_0, 1 µM MnSO_4_⸳H_2_O, 0.005 µM (NH_4_)_6_MO_7_O_24_⸳4H_2_O, 2498.41 µM Ca(NO_3_)_2_⸳4H_2_O, 499.7 µM KH_2_PO_4_, 1000.12 µM MgSO_4_⸳7H_2_O, 2504.70 µM KNO_3_, and 100 µM Na Fe⸳EDTA at 22 ℃/27 ℃ during 8/16 h dark/light periods, respectively, with a light intensity of 180 μmol⸳m^−2^⸳s^−1^ and 60% relative humidity. After 15 days, equally sized cotton seedlings were transferred into the nutrient solution with different Cu^2+^ concentrations. The treatment concentrations were composed of 0 (without addition of Cu^2+^), 0.2 (control group), 50, 100, and 200 μM Cu^2+^, supplied as CuSO_4_⸳5H_2_O. The nutrient solutions were continuously renewed every 4 days, and then the pH value was modified to 5.6–5.7 with 0.1 M HCl or 0.1 M NaOH. Plants were harvested after 10 days of Cu^2+^ treatment for future analysis.

### 2.2. Determination of Growth Parameters

Growth parameters of the cotton seedlings were evaluated by determining leaf area, root length, and shoot length. The leaf area of seedlings was determined by the area-weighing method, and the lengths of roots and shoots were recorded using a meter ruler.

### 2.3. Measurement of Photosynthetic Pigment Contents

Chlorophyll and carotenoid concentrations were determined according to Lichtenthhaler et al., 1983 [27], with a few modifications. Briefly, the top-most expanded leaves were randomly cut and soaked in 80% acetone in a ratio of 1:10 *w/v* until the pigments were completely extracted and the leaf became colorless. The process was performed in darkness. The extracts were centrifuged for 15 min at 4000× *g* to remove any residues. The supernatant was measured at 663, 645, and 470 nm using a visible spectrophotometer v-1000 (AoYi Instrument Co., Ltd., Shanghai, China) for chlorophyll a, chlorophyll b, and carotenoids, respectively, and 80% acetone was used as a blank control. 

### 2.4. Determination of Copper Content

For Cu^2+^ content analysis, the root and leaf samples were harvested separately, rinsed with tap water, and immersed in 20 mM Na_2_-EDTA for 15 min to remove any trace elements adhering to the tissue. The root and leaf samples were oven-dried at 75 °C for 48 h. Dried samples (0.1 g) were ground and acid-digested with an HNO_3_ mixture for 24 h at 80 °C, followed by Cu^2+^ estimation using an atomic absorption spectrophotometer (Perkin Elmer-model 2380, C.R.G.R.) [28].

### 2.5. Detection of H_2_O_2_ and MDA Levels

The accumulation of H_2_O_2_ in leaves was measured by monitoring the A415 of the titanium–peroxide complex, as previously described by Liu et al., 2018 [29], with some modifications. Absorbance values were calibrated to a standard curve generated with known concentrations of H_2_O_2_. Recovery was checked by adding various amounts of H_2_O_2_ to the leaf extracts as an internal standard. The level of lipid peroxidation was determined according to Thounaojam et al., 2012 [2]. Fresh leaves (0.2 g) were homogenized with 5 mL of 0.25% TBA. The homogenate was boiled for 30 min at 95 °C and centrifuged at 10,000× *g* for 10 min. The absorbance of the supernatant was recorded at 532 nm and 600 nm using a visible spectrophotometer 722N (Yidan Analytical Instrument Co., Ltd., Shanhai, China) with an extinction coefficient of 155 mM^−1^·cm^−1^.

### 2.6. Measurement of POD Activity and GSH Content

Frozen leaves were homogenized and centrifuged, and then the supernatant was immediately used for the antioxidant enzyme assays. The POD activity was measured by guaiacol oxidation prior to carrying out the method described by Zhang et al., 2016 [30]. To determine the GSH content, samples (0.5 g) were crashed with 5 mL of 10% (*w*/*v*) TCA, and the homogenate was centrifuged at 15,000× *g* for 15 min at 4 °C. The GSH content was determined according to the method previously described by Ates et al., 2009 [31].

### 2.7. Determination of Soluble Sugar Content

Soluble sugar content was conducted similarly to the method previously explained by Anjorin et al., 2016 [32], with a few modifications. Fresh leaves (0.5 g) were homogenized in 80% ethanol and then incubated at 75 °C for 10 min. Next, 40 mL of the supernatant were mixed with 80% carbolic acid and 4 mL of concentrated sulfuric acid, and then the absorbance was recorded at 490 nm using a visible spectrophotometer 722N (Yidan Analytical Instrument Co., Ltd., Shanhai, China). The concentration of soluble sugar was determined by a calibration curve prepared from a sucrose solution and was expressed as mg·g^−1^ FW.

### 2.8. Statistical Analysis

All statistical analyses were performed using the SPSS 21.0 computer software package. Data for each treatment were expressed as mean values ± S.D., with three replicates. Differences among the groups were examined by one-way ANOVA followed by LSD. *p* < 0.05 was considered statistically significant.

## 3. Results

### 3.1. Plant Growth Conditions under Cu^2+^ Stress

**Figure 1:**
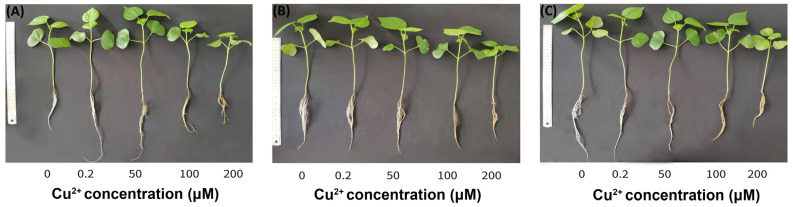
Effects of different Cu^2+^ concentrations on cotton morphology: (**A**) 9053; (**B**) sGK9708; (**C**) Zhongmian 63.

The effect of the different Cu^2+^ concentrations on the growth of cotton seedlings is presented in Figure 1. The average data based on plant growth showed that the parental line sGK9708 and the hybrid variety Zhongmian 63 were more tolerant to Cu^2+^ than the maternal genotype 9053. Although the toxicity of Cu^2+^ greatly inhibited the growth of the cotton seedlings, the plant height and leaf area of the three cotton genotypes showed strong tolerance when the Cu^2+^ concentration was 50 μM, compared with the control group (Figure 1A,B), but the growth of the roots was inhibited (Figure 1C). This suggested that the roots are more vulnerable to Cu^2+^ stress than the shoots.

### 3.2. Content of Photosynthetic Pigments

The toxicity of Cu^2+^ severely reduced the content of chlorophyll a and chlorophyll b in the cotton seedling leaves (Table 1). In the absence of Cu^2+^, the chlorophyll content of the three genotypes of cotton seedlings decreased compared with that of the control group. When Cu^2+^ was in excess, the chlorophyll content of the three genotypes of cotton seedlings also decreased compared to that of the control group. On the contrary, the chlorophyll contents of sGK9708 and Zhongmian 63 temporarily increased when the Cu^2+^ concentration was 50 μM, significantly so for Zhongmian 63, but subsequently decreased as the Cu^2+^ concentration increased. The ratio of chlorophyll-a content to chlorophyll-b content tended to reduce with increasing Cu^2+^ concentrations in the nutrient solution. This indicated that the content of chlorophyll-a decrease was higher than that of chlorophyll-b.

### 3.3. Determination of Cu^2+^ Content

Under Cu^2+^ stress, different cotton varieties exhibited differences in Cu^2+^ uptake and transport (Table 2). Although the absorption of Cu^2+^ was different, the trends of the Cu^2+^ accumulation in different tissues of all three varieties were similar. Under different Cu^2+^ treatments, the Cu^2+^ concentrations were highest in the roots, then in the leaves, and finally in the stems. However, the highest accumulation of Cu^2+^ was found in Zhongmian 63, followed by that of sGK9708 and 9053 (Table 2). In comparison to the parent lines, the quantity of Cu^2+^ transport from the roots to the shoots was lower in Zhongmian 63. This indicated that Zhongmian 63 is more capable of enriching Cu^2+^ in the roots, effectively reducing the toxicity of Cu^2+^ to the cotton seedlings.

### 3.4. Determination of Morphological Characters and Soluble Sugar Content

**Figure 2:**
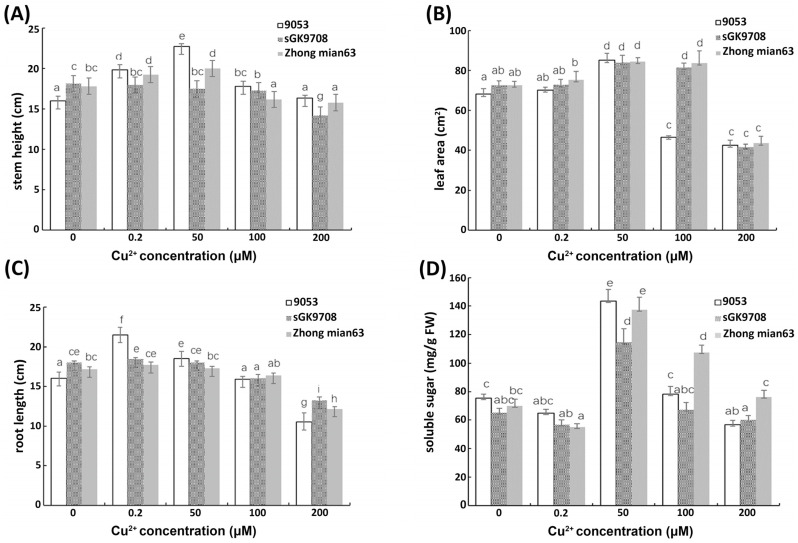
Effect of different Cu^2+^ concentrations on plant growth of three cotton genotypes after the 10th day of treatment: (**A**) stem height; (**B**) leaf area; (**C**) root length; (**D**) soluble sugar. The data are the mean of three separate experiments ± S.D. (*n* = 3). Each bar represents the mean ± SD. Different lowercase letters above the same column indicates a significant difference between columns at (*p* < 0.05 according to multiple comparisons by the LSD test), while a same letter indicates no significance difference.

Morphological changes, including stem height, root height, and leaf area, were measured in response to different Cu^2+^ concentrations. As shown in Figure 2, cotton stem and root height increased in all three genotypes at 0, 0.2, and 50 µM but decreased as Cu^2+^ concentration increased from 100 to 200 µM (Figure 2A,C). The cotton leaf area responses to Cu^2+^ showed a similar trend, and the 9053 variety showed an extreme decrease in leaf area at 100 and 200 µm (Figure 2B). Regarding the effects of different concentrations of Cu^2+^ on the soluble sugar content of all three cotton genotypes, the results showed that the soluble sugar content in leaves reached its maximum at a 50 μM Cu^2+^ concentration and then decreased with further increases in the Cu^2+^ concentration. At Cu^2+^ concentrations of 100 μM and 200 μM, the soluble sugar content of Zhongmian 63 was significantly higher than that of the parent lines (Figure 2D).

### 3.5. Determination of H_2_O_2_ and MDA Content and Antioxidant Scavenging Ability

**Figure 3:**
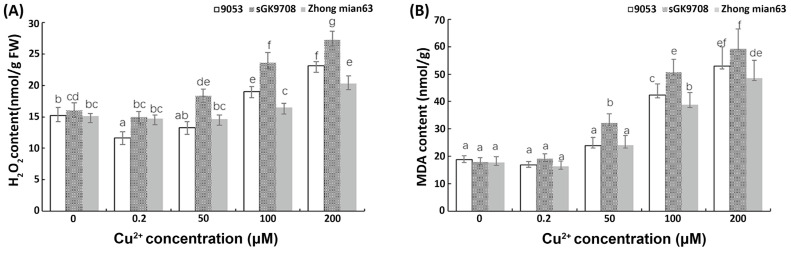
Effect of different Cu^2+^ concentrations on the contents of H_2_O_2_ and MDA in leaves of three cotton genotypes. (**A**) H_2_O_2_ concentration; (**B**) MDA content. The data are the mean ± S.D. (*n* = 3). Each bar represents the mean ± SD. Different lowercase letters above the same column indicates a significant difference between columns at (*p* < 0.05, Tukey’s test), while a same letter indicates no significance difference.

**Figure 4:**
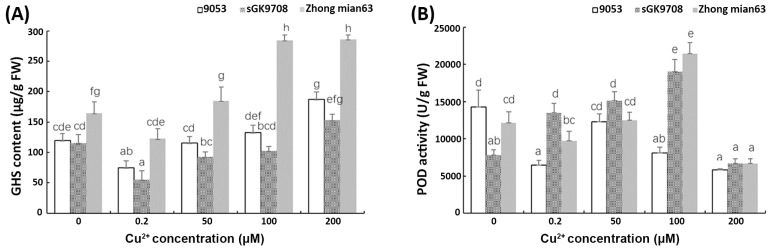
Antioxidant scavenging enzyme in the leaves of three cotton genotypes: (**A**) GSH content; (**B**) POD activity. The data are the mean ± S.D. (*n* = 3). Each bar represents the mean ± SD. Different lowercase letters above the same column indicates a significant difference between columns at (*p* < 0.05, Tukey’s test), while a same letter indicates no significance difference.

The leaf H_2_O_2_ and MDA content of the three genotypes increased with an increasing Cu^2+^ concentration (Figure 3). At a Cu^2+^ concentration of 200 μM, the H_2_O_2_ and MDA in the three cotton genotypes reached the highest levels. However, compared with the parent lines, the H_2_O_2_ and MDA content in the Zhongmian 63 leaves increased slowly with increasing Cu^2+^ concentrations (Figure 3A,B). This indicated that Zhongmian 63 has a substantial ability to scavenge ROS compounds. The leaf GSH content of the three genotypes increased with increasing Cu^2+^ concentrations (Figure 4A). When Cu^2+^ was excessive, the GSH content increased with the increase in Cu^2+^ concentration in the three cotton genotypes. At the Cu^2+^ concentration of 200 μM, the GSH content in the leaves of the three cotton genotypes also reached the highest levels. However, under the conditions of different Cu^2+^ concentration treatments, the GSH content in the Zhongmian 63 leaves was significantly higher than that of the parent lines. Generally, regarding Cu^2+^, POD activity in the leaves of the cotton seedlings first increased and then decreased (Figure 4B). In 9053, POD activity in the leaves of seedlings peaked at a Cu^2+^ concentration of 50 μM and then declined significantly. Similarly, at Cu^2+^ concentrations of 100 μM, sGK9708 and Zhongmian 63 had extremely high POD scavenging activities, with higher expression in Zhongmian 63. Lastly, POD activity decreased significantly when the Cu^2+^ concentration reached 200 μM.

## 4. Discussion

### 4.1. Morpho-Physiological Changes and Copper Absorption

In recent decades, increasing modern agricultural practices, anthropogenic activities, rapid industrialization, rapid urbanization, and burning of fossils fuels have amplified the threshold level of various heavy metals in the plant–soil complex, especially in aquatic environments, causing negative impacts on the living forms [33,34]. Much land under plant cultivation faces heavy metal contamination because of the unlimited and excessive usage of chemical agriculture products (chemical manures, pesticides, fertilizers, weedicides, and herbicides), mining, urban and rural municipal solid waste, metalliferous mines, energy and fuel production, mining, the mismanagement of agribusiness wastewater, power transmission, and industrial sources, resulting in heavy metal release [35,36,37]. Over the last 1000 years, the increased use of Cu^2+^ as a pesticide has become one of the world’s major environmental problems. According to previous studies, the management of Cu^2+^ as a fungicide has always been a part of agriculture [25]. However, Cu^2+^ does not degrade as quickly as it accumulates in soil, which causes elevated levels that can reach up to 3000 mg kg^−1^in agricultural areas [38]. Yang et al., 2015 [39], proposed that Cu^2+^ toxicity largely affects root growth and morphology in rice and maize and tends to accumulate in the root tissue, which can be transferred to the shoots. Studies on the effects of heavy metal pollutants on the physiological and biochemical characteristics of plants and remedial techniques have been well-documented. For instance, Bouazizi et al. 2010 showed that CuSO_4_ causes a reduction in biomass and leaf number in *Phaseolus vulgaris*. Ref. [28] showed that CuSO4 reduces the biomass and leaf number in *Phaseolus vulgari*. Moreover, existing research shows that CuSO4 toxicity reduces Cu^2+^ growth rates and necrosis as well as stunted growth in woody ornamentals [25,40]. Further studies proved that Cu^2+^ accumulation also affects plant morphology by lowering the stem height of rice seedlings and the chlorophyll content of cotton roots [2,20]. In the present study, a decrease in cotton morphological growth under an increasing level of Cu^2+^ indicated a Cu^2+^ toxicity effect at elevated concentrations on cotton roots, leaves, and stems (Figure 2A–C). The hybrid genotype, Zhongmian 63, was more tolerant to Cu^2+^ than the parent lines (9053 and sGK9708). We hypothesized that growth retardation might be due to a significant reduction in oxidative stress and probably the photosynthetic pigments in the leaves. Chlorophyll is considered an important biomarker of abiotic stress, including from heavy metals [41]. The existing research proved that excess Cu^2+^ is capable of disrupting the chloroplast and thylakoid membrane composition, triggers oxidative stress in plant cells, and reduces stomatal conductance and declining photosynthetic gas exchange and chlorophyll fluorescence parameters [42,43]. In addition, Cu^2+^ concentrations significantly affect the expression levels of the chlorophyll-a and carotenoid content of the phytoplankton *Nitzschia sp*. [44]. Furthermore, other previous studies demonstrated that elevated Cu^2+^ concentrations not only cause chlorosis, which reduces plant biomass, but also interfere with the electron transport chain system by saturating the protein pigment plastocyanin, which is involved in photosynthesis [34]. Similarly, in arable crops, such as rice and tomatoes, higher Cu^2+^ concentrations cause chlorophyll reduction [45,46]. In *Urtica dioica* and *Fallopia japonica,* the chlorophyll content slightly increased with increasing Cu^2+^ [25]. A recent report on lentil plants showed a significantly reduced chloroplast pigment composition, including chl a and chl b, along with carotenoids, at high Cu^2+^ concentrations (3.0 mM) [47]. Similarly, in the present investigation, the chlorophyll content in the leaves of cotton seedlings decreased with increasing Cu^2+^ concentration (Table 1). However, the carotenoid content increased under Cu^2+^ stress conditions compared to that of the control group (Table 1). From previous studies, carotenoids are known to increase under Cu^2+^ toxicity and act as biological indicators of heavy metal effects in cyanobacteria [48]. We speculate that the decrease in chlorophyll content and increase in carotenoid content in cotton genotypes may be a signal of Cu^2+^ toxicity. Heavy metal toxicity in plants may result from the increasing contents in the soil, which directly affects the quality and safety of agricultural products [49]. Cotton is well-known for alleviating poor soil conditions by adsorbing polluting heavy metals, such as Cu^2+^, Ag, Au and Pb, in farmland [20]. Nevertheless, reproductive processes are greatly inhibited at higher concentrations [50]. According to previous studies, high concentrations of Cu^2+^ lead to plant root toxicity, causing root stunting, which directly interferes with the root’s capacity to absorb the nutrients delivered by diffusion [51]. In the present study, cotton roots had the highest level of Cu^2+^ absorption, followed by leaves and stems (Table 2). In addition, at a CuSO_4_ level of 200 µM, cotton roots were stunted in all three genotypes. We hypothesized that root stagnation development in cotton roots could be the result of a high accumulation of Cu^2+^ (Figure 1). 

### 4.2. ROS Accumulation Levels and Peroxidation 

The production of ROS is a common phenomenon of stress, and ROS attack polyunsaturated fatty acids and lead to lipid peroxidation [52]. Chronically overexposing plants to copper increases the production of reactive oxygen species, causing the accumulation of different ROS such as superoxide (O_2_•−), hydrogen peroxide (H_2_O_2_), and hydroxyl radicals (•HO), which disrupt redox homeostasis and cause oxidative damage to cells at the lipid, protein, and nucleic acid levels [51,53,54]. Many studies showed that heavy metal toxicity causes significant accumulation of H_2_O_2_ and MDA [45,55]. For instance, in wheat, long-term exposure to a high level of CuSO_4_ (75 µM) influenced ROS generation [51]. Moreover, in *M. sativa* seedlings, excess Cu^2+^ increased ROS production by destroying the H_2_O_2_ scavenging system [29,56]. Similarly, in fenugreek, Cu^2+^ toxicity induced an increase in the rate of hydrogen peroxide production and lipid peroxidation, displaying oxidative stress [24]. MDA concentrations in *Glycine max* and *Lupinus albus L*. were reported to increase in root nodules when exposed to a high level of CuSO_4_ (192 µM) [57]. In this study, increasing the CuSO_4_ concentration in the culture medium led to an increase in H_2_O_2_ and MDA in the leaves of cotton seedlings at values of 100 and 200 µM in all the cotton genotypes, with higher levels in the 9053 and sGK9708 varieties (Figure 3). We hypothesized that high concentrations of ROS and MDA may be a signal of oxidative stress activation, enzymatic inactivation, and lipid peroxidation [58].

### 4.3. Antioxidant Alleviation and Soluble Sugar Defensive Mechanisms

At the cellular level, plants protect their cells from oxidative stress through a range of biochemical mechanisms (i.e., enzymatic and non-enzymatic antioxidants) [59]. Plant enzymatic defenses comprise antioxidant enzymes (catalase, ascorbate peroxidase (APX), phenol peroxidase (POX), glutathione peroxidase (GPX), and superoxide dismutase), together with non-enzymatic substances (proline, GHS, and ascorbate (ASA)), which promote the scavenging of ROS [20,34,60]. According to previous reports, high Cu^2+^ levels induce the activities of antioxidant enzymes (SOD, CAT, and POD) as well as non-enzymatic antioxidants (GSH) [51]. In the present study, both enzymatic and non-enzymatic defense mechanisms were stimulated in response to increased Cu^2+^ levels (Figure 4). POD plays a major role in the H_2_O_2_ scavenging enzymes that remove H_2_O_2_ from chloroplasts and the cytosol of plant cells [61]. The role of POD scavenging in plants against heavy metal toxicity was proven to increase and decrease as metal toxicity increases [56,62]. The enhancement of peroxidase (POD) activities was reported in the roots and leaves of cotton genotypes exposed to Cu^2+^ (100 mg kg^−1^) [22]. Application of Cu^2+^ in *Linum usitatissimum* resulted into SOD and POD activities’ enhancement in relation to increasing Cu^2+^ concentrations [17]. Similarly, in this study, POD antioxidant activity first increased at 0, 0.2, 50, and 100 µM but then significantly declined at 200 µM. We speculate that the POD fluctuations caused by Cu^2+^ concentrations might be a biomarker for heavy metals or lipid peroxidation [63]. GSH acts as an antioxidant and detoxifies H_2_O_2_ via the ascorbate–glutathione cycle. Most importantly, it acts as a precursor of phytochelatins (PCs) by chelating Cu^2+^ and participating in detoxification and tolerance [20]. Wang et al., 2023 [23], proposed that Cu2^+^-induced chlorosis in Arabidopsis seedlings could be mitigated by the exogenous application of glutathione (GSH). Cu^2+^-induced elevation in GSH content was observed in rice [2]. In addition, the toxicity of cadmium (Cd) and other heavy metal ions is primarily reduced by PC complexation activity [19,64]. In this work, Cu^2+^ levels of 50, 100, and 200 µM dramatically increased the GSH content in Zhongmian 63 (Figure 4A). We hypothesized that GHS may play a key role in metal chelation and removal under the condition of excess Cu^2+^ content in cotton seedlings [13]. Soluble sugars were declared to take part in the control of photosynthetic activity, sensing, and the control of ROS balance [65]. Li et al., 2020 [20], explained that plant cells usually accumulate soluble sugars to diminish intracellular osmotic potential and guarantee the normal supply of water under heavy metal stress, in order to maintain normal physiological functioning. In the present work, the content of soluble sugar in the leaves of cotton seedling first increased and then decreased with the increase in the Cu^2+^ concentration (Figure 2D). We speculated that a lower Cu^2+^ concentration accelerates the decomposition of high-density carbohydrates in plants and inhibits their synthesis. Therefore, photosynthetic products directly form low molecular mass substances, such as sucrose, resulting in soluble sugar accumulation. On the contrary, at high Cu^2+^ concentrations, the anabolism and growth of plants were repressed, and the photosynthetic capacity of the plants reduced, resulting in a decrease in soluble sugar content. Hence, Cu^2+^ toxicity significantly inhibits soluble sugar content, affecting cotton tolerance. 

Heavy metals arise from many sources, such as industry, mining, and agriculture. However, sources in the agricultural sector can be categorized into fertilization, pesticides, livestock manure, and wastewater [66]. Heavy metal pollution in the environment has been rapidly expanding and causing havoc, particularly in the agricultural sector, by accumulating in soil and plant uptake [67]. Cu^2+^ accumulation can be useful for plant growth and development but is extremely toxic to plant seedlings. Previous studies showed that Cu^2+^ toxicity has a less significant effect on maize [68]. Nonetheless, Cu^2+^ in higher contents had a damaging impact on the growth as well as root morphology of plants [11]. Cotton can withstand adverse environmental conditions in several phases and through phytoremediation [69]. However, seedlings may have a different response to heavy metal toxicity. As observed in this study, the morphological effect of Cu^2+^ shows no significant changes in plant leaves; however, at extreme concentrations, the cotton root morphology exhibits a declination in growth. We argue that plants may have specific threshold points for metal toxicity, but levels above the desired threshold may be detrimental to growth and development. This depicts the notion that the agricultural usage of heavy metals such as Cu^2+^ may be useful for plants, but excessive usage resulting in high soil accumulation may cause toxicity thresholds above normal, which may affect plants at tender growth stages. Hence, we recommend that a deficiency of Cu^2+^ in cotton seedlings may be solved by applying Cu^2+^-encompassing fertilizers to the soil, but high Cu^2+^ levels can be managed by reducing Cu^2+^-related fertilizer application during the seedling’s growth stages [70]. We further suggest cotton seedlings be cultivated in a controlled environment (hydroponic culture), which may also be effective for cotton growth, since varying soil copper concentrations may be monitored and adjusted until fully mature to withstand adverse environmental conditions before being exposed to the outside environment.

In summary, this study demonstrated that an excessive Cu^2+^ concentration severely restricts the root, shoot, leaf, and photosynthetic parameters of cotton seedlings. However, the cotton seedlings showed strong tolerance to a range of Cu^2+^ concentrations. Higher levels of Cu^2+^-induced oxidative stress increased the H_2_O_2_ production in cotton seedlings. On the other hand, when compared with the parent line, the hybrid lines effectively increased their tolerance to Cu^2+^ by increasing GSH content, POD activity, and soluble sugar content and reducing the transfer of Cu^2+^ to the ground.

## Figures and Tables

**Table 1:** Effect of Cu^2+^ treatments on the contents of chlorophyll and carotenoid in cotton seedlings.

Cotton Variety	Cu^2+^ Concentration (μM)	Chlorophyll a (mg/g FW)	Chlorophyll b (mg/g FW)	Chlorophyll (a + b) (mg/g FW)	Chlorophyll a/b	Carotenoid (mg/g FW)
9053	0	0.28 ± 0.01 bcd	0.08 ± 0.01 b	0.36 ± 0.02 bcd	3.36 ± 0.14 c	0.051 ± 0.003 bc
	0.2	0.33 ± 0.05 cd	0.09 ± 0.02 bc	0.43 ± 0.06 cd	3.52 ± 0.09 c	0.048 ± 0.006 ab
	50	0.30 ± 0.04 cd	0.09 ± 0.02 bc	0.39 ± 0.06 cd	3.12 ± 0.09 b	0.045 ± 0.004 ab
	100	0.27 ± 0.03 bcd	0.08 ± 0.01 b	0.35 ± 0.04 bcd	3.26 ± 0.06 bc	0.049 ± 0.007 abc
	200	0.11 ± 0.01 a	0.04 ± 0.01 a	0.16 ± 0.01 a	2.64 ± 0.21 a	0.049 ± 0.004 abc
Zhongmian 63	0	0.24 ± 0.02 bcd	0.08 ± 0.01 b	0.31 ± 0.02 bcd	3.06 ± 0.20 c	0.044 ± 0.003 ab
	0.2	0.25 ± 0.04 bc	0.08 ± 0.02 b	0.32 ± 0.05 bc	3.06 ± 0.11 b	0.038 ± 0.004 a
	50	0.35 ± 0.06 d	0.11 ± 0.02 c	0.46 ± 0.08 d	3.15 ± 0.03 b	0.050 ± 0.005 bc
	100	0.28 ± 0.10 cd	0.09 ± 0.03 bc	0.37 ± 0.13 bcd	3.11 ± 0.10 b	0.048 ± 0.016 abc
	200	0.19 ± 0.02 ab	0.07 ± 0.01 b	0.26 ± 0.03 ab	2.67 ± 0.07 a	0.055 ± 0.006 bc
sGK9708						
	0	0.30 ± 0.03 cd	0.10 ± 0.02 bc	0.40 ± 0.04 cd	3.09 ± 0.17 bc	0.050 ± 0.003 bc
	0.2	0.32 ± 0.08 cd	0.09 ± 0.03 bc	0.42 ± 0.11 cd	3.37 ± 0.03 bc	0.044 ± 0.005 ab
	50	0.34 ± 0.06 cd	0.10 ± 0.02 bc	0.43 ± 0.08 cd	3.39 ± 0.13 bc	0.068 ± 0.004 abc
	100	0.29 ± 0.03 cd	0.09 ± 0.01 bc	0.38 ± 0.04 bcd	3.13 ± 0.09 b	0.049 ± 0.003 abc
	200	0.17 ± 0.08 a	0.07 ± 0.02 b	0.24 ± 0.1 ab	2.34 ± 0.4 a	0.058 ± 0.007 c

Each column represents the mean ± SD. Different lowercase letters in the same column represent the significant differences at (*p* < 0.05, Tukey’s multiple comparison).

**Table 2:** The Cu^2+^ contents in the leaf, stem, and root of cotton under different Cu^2+^ concentrations.

Cotton Variety	Cu^2+^ Concentration (μM)	Leaf (μg/g DW)	Stem (μg/g DW)	Root (μg/g DW)
9053	0	8.67 ± 0.85 a	4.43 ± 0.70 a°	20.27 ± 1.46 a
	0.2	10.40 ± 1.20 a	5.33 ± 0.35 a	22.20 ± 2.00 a
	50	28.95 ± 3.13 b	8.70 ± 1.45 bc	121.67 ± 5.8 b
	100	46.27 ± 4.76 c	15.87 ± 2.06 e	262.87 ± 23.1 de
	200	75.54 ± 11.40 e	25.37 ± 2.80 g	386.30 ± 31.96 f
Zhongmian 63	0	7.60 ± 0.36 a	4.47 ± 0.70 a	20.47 ± 1.74 a
	0.2	8.83 ± 0.81 a	5.43 ± 0.59 a	24.77 ± 3.32 a
	50	23.79 ± 2.36 b	9.03 ± 0.83 c	158.63 ± 10.92 bc
	100	39.43 ± 5.80 c	13.60 ± 1.55 de	286.20 ± 17.76 de
	200	65.07 ± 4.73 d	20.57 ± 2.41 f	489.20 ± 32.03 g
sGK9708	0	8.50 ± 0.70 a	5.63 ± 0.45 ab	18.57 ± 1.12 a
	0.2	9.47 ± 1.20 a	6.30 ± 0.50 abc	20.07 ± 2.14 a
	50	30.27 ± 1.99 b	12.40 ± 2.05 d	139.83 ± 10.63 bc
	100	43.53 ± 3.56 c	20.27 ± 1.96 f	253.00 ± 10.50 d
	200	76.97 ± 8.06 e	33.90 ± 3.60 h	475.73 ± 24.83 g

Each column represents the mean ± SD. Different lowercase letters in the same column represent the significant differences at (*p* < 0.05, Tukey’s multiple comparison).

## Data Availability

Not applicable.

## Abbreviations

Cu^2+^	copper(II)
MTs	Metallothioneins
PCs	Phytochelatins
SOD	superoxide dismutase
CAT	catalases
POD	Peroxidase
GSH	Glutathione
AsA	ascorbate
ROS	reactive oxygen species
MDA	Malondialdehyde
